# Impact of robust optimization and beam configuration on the distribution stability of LET under delivery uncertainties in carbon‐ion therapy

**DOI:** 10.1002/acm2.70878

**Published:** 2026-10-27

**Authors:** Wisawa Phongprapun, Mutsumi Tashiro

**Affiliations:** ^1^ Department of Medical Physics for Heavy Ion Therapy Graduate School of Medicine Gunma University Maebashi Gunma Japan; ^2^ Gunma University Heavy Ion Medical Center Maebashi Gunma Japan

**Keywords:** carbon‐ion radiotherapy, linear energy transfer, low‐dose‐averaged LET, phantom‐based study, planning target volume

## Abstract

**Background:**

Carbon‐ion radiotherapy exploits high linear energy transfer (LET) to enhance biological effectiveness, and low dose‐averaged LET (LETd) within the target volume is associated with local recurrence in pancreatic cancer. Although robust optimization is widely used to improve dose robustness against delivery uncertainties, systematic investigation of LET robustness across optimization strategies and beam configurations remains scarce.

**Purpose:**

To evaluate the robustness of LET metrics in carbon‐ion therapy under delivery uncertainties using a phantom‐based design, comparing planning target volume (PTV)‐based with robust optimization and assessing the impact of seven beam configurations, and to characterize the differential response of LET and clinical dose (relative biological effectiveness [RBE]‐weighted dose) metrics.

**Methods:**

A 30 × 30 × 30 cm^3^ water‐equivalent phantom with rod‐ and C‐shaped target geometries was planned in RayStation 12A with 50 Gy (RBE) in 10 fractions. Section 1 used two opposing lateral fields to compare PTV‐based optimization (1, 3, 5 mm margins) with robust optimization incorporating translation, density, and combined uncertainties. Section 2 evaluated seven beam configurations, ranging from two‐field to four‐field arrangements, under PTV‐based (3 mm) and robust (± 3%/3 mm) optimization. LETd98%, LETd mean, and LETd2% were extracted for the clinical target volume and organs at risk (OARs). Robustness was quantified using averaged interquartile range (AVG.IQR) and sensitivity slopes from 1% to 5% density uncertainty (S1→5), and configurations were ranked by minimal‐change and biologically favorable criteria. Spot weight distributions were analyzed to examine the mechanistic basis of the observed differences.

**Results:**

Density‐related uncertainties dominated LET variability, with LETd2% the most sensitive metric (AVG.IQR up to 40% for C‐shaped targets). Robust optimization with combined translation‐density parameters achieved lower variability than PTV‐based optimization. LET and RBE‐weighted dose metrics responded divergently, with the LETd mean increasing while the mean clinical dose decreased under density perturbations. Beam direction significantly affected LETd2% variability (*p* < 0.001 for all configurations). Two lateral fields (2Lat) ranked highest under the minimal‐change criterion, whereas four‐field oblique (4FO) with robust optimization ranked first under the biologically favorable criterion, balancing increased target LET against reduced OAR exposure. Spot weight analysis showed that, for the rod‐shaped target, only robustness scenarios including density uncertainty used more energy layers and shifted weight away from distal layers (distal‐third weight fraction 56.4–57.4% vs. 60.8% for PTV‐based optimization); this pattern was not reproduced for the C‐shaped target.

**Conclusions:**

Density uncertainties predominantly govern LET variability in carbon‐ion therapy, with LETd2% the most sensitive indicator, and redistribution of spot weight away from distal energy layers provides a plausible mechanism. Beam configuration selection should reflect clinical priorities: 4FO with robust optimization when biological effectiveness is prioritized, and 2Lat for physical LET stability. These phantom‐based findings support explicit LET robustness evaluation in carbon‐ion treatment planning and warrant validation in patient studies.

## 1 INTRODUCTION

Carbon‐ion radiotherapy (CIRT) offers distinct physical and radiobiological advantages over conventional photon therapy.^1^, ^2^ The sharp Bragg peak with reduced lateral scattering enables highly conformal dose delivery while sparing normal tissues,^3^ driving worldwide clinical adoption and technological advancement.^4^ Linear energy transfer (LET), a key radiobiological quantity in particle therapy, varies along the beam path and strongly correlates with biological effects.^5^ In carbon‐ion beams, high LET near the distal edge is associated with higher relative biological effectiveness (RBE),^6^, ^7^ and recent γH2AX foci analysis supports incorporating LET data in treatment planning.^8^

However, the existing clinical implementations of CIRT still have not fully used the active biological properties of carbon ions.^9^ From the point of view of treatment planning, the dose‐averaged LET (LETd) distribution within the target volume may not always be evenly distributed and is not optimal for large target volumes, where central areas generally receive lower LETd values.^10^, ^11^ These heterogeneous LETd distributions are associated with decreases in tumor control probability at specific tumor sites, such as pancreatic cancer.^12^, ^13^ Notably, Sakamoto et al.^13^ recently demonstrated in the largest clinical cohort to date (106 patients) that low LETd within the clinical target volume (CTV) was significantly associated with local recurrence in pancreatic cancer treated with CIRT (CTV Lmin cutoff: 42.2 keV/µm, *p* = 0.030), providing robust clinical evidence for the importance of LET optimization. These findings have been noted to drive the increasing consideration of LET and absorbed doses in treatment planning strategies during optimization.^14^

Classical treatment planning in particle therapy was based on the planning target volume (PTV) concept, where geometric margins with respect to CTV were included to incorporate setup uncertainties.^15^ Although successful in photon therapy, this principle has limited applicability in particle therapy owing to the pronounced sensitivity of proton and carbon‐ion dose distributions to range and setup uncertainties.^15^, ^16^ Even with beam‐specific PTV, which applies asymmetric margins along the beam direction to account for range variations,^17^ margin‐based methods do not fully compensate for range uncertainties that can cause beam under‐ or overshooting, resulting in reduced target coverage and increased dose to normal tissues.^18^ Hence, robust optimization is an alternative approach that explicitly incorporates uncertainty scenarios within the optimization process.^18^, ^19^ Robust optimization yields better stability in target coverage and organ‐at‐risk sparing in the face of the uncertainties related to setup and range than traditional PTV‐based optimization.^20^, ^21^, ^22^ Although the robustness of the dose for particle therapy has received extensive attention, optimization strategies have not received significant attention for improving LET distributions while optimizing dose uncertainty. This limitation is particularly interesting considering the well‐known radiobiological importance of LET.^23^ Recent attempts to propose the LET trilemma have highlighted the inherent trade‐offs in carbon‐ion therapy: achieving high target LETd often comes at the cost of reduced dose homogeneity or compromised robustness against range uncertainties.^24^ Characterizing these competing factors will be critical for the development of treatment planning frameworks that balance dosimetric robustness and biological considerations across a spectrum of approaches. Moreover, beam configuration is an essential planning parameter that affects dose and LET characteristics.^25^ Varying beam angles affect CIRT robustness. Zhou et al. comprehensively reviewed beam‐angle selection strategies in particle therapy, highlighting the importance of angle optimization for plan quality and robustness.^26^ Previous studies have investigated water‐equivalent thickness‐based beam selection^27^ and adaptive beam angle strategies for pancreatic cancer,^28^ and oblique beam configurations have been shown to increase robustness compared to traditional lateral arrangements in prostate cancer.^29^ However, systematic studies comparing LET variation across different beam configurations and optimization strategies remain scarce.

Therefore, this study aimed to systematically evaluate the robustness of LET metrics in carbon‐ion therapy under delivery uncertainties. A phantom‐based approach was selected to provide controlled conditions, isolate individual variables, and enable direct comparisons across planning scenarios. The objectives were to (1) compare PTV‐based and robust optimization strategies incorporating translation, density, and combined uncertainties; (2) assess the impact of seven beam configurations on LET stability; and (3) characterize the differential response between LET and clinical dose (RBE‐weighted dose) metrics. Using rod‐ and C‐shaped target geometries, we developed a ranking system to identify the optimal beam configurations for clinical treatment planning.

## 2 MATERIALS AND METHODS

### 2.1 Phantom design

A 30 × 30 × 30 cm^3^ cubic phantom with a uniform Hounsfield unit value of 0 was generated using Python 3.11.7 to simulate water‐equivalent tissue. Two gross tumor volume (GTV) geometries were created at the phantom center: a rod‐shaped GTV (diameter, 5 cm) and a C‐shaped GTV (width, 2 cm) containing a rod‐shaped organ at risk (OAR) (diameter, 2 cm) at the center of the concavity. The CTV was generated by applying a 5‐mm expansion margin to the GTV. For PTV‐based optimization, three PTVs were created using CTV expansion margins of 1, 3, and 5 mm (Figure 1). These expansions follow clinical practice, with the 5‐mm GTV‐to‐CTV margin corresponding to that used in carbon‐ion protocols for pelvic tumors^30^ and the three PTV margins spanning conventional, image‐guided, and stereotactic‐accuracy scenarios, each matched to a robust optimization scenario of equivalent magnitude. All contours were delineated using a RayStation 12A (RaySearch Laboratories, Stockholm, Sweden). A modified Microdosimetric Kinetic Model (MKM)^31^, ^32^ with the Human Salivary Gland (HSG) cell type^33^ was used for biological dose calculations.

**FIGURE 1:**
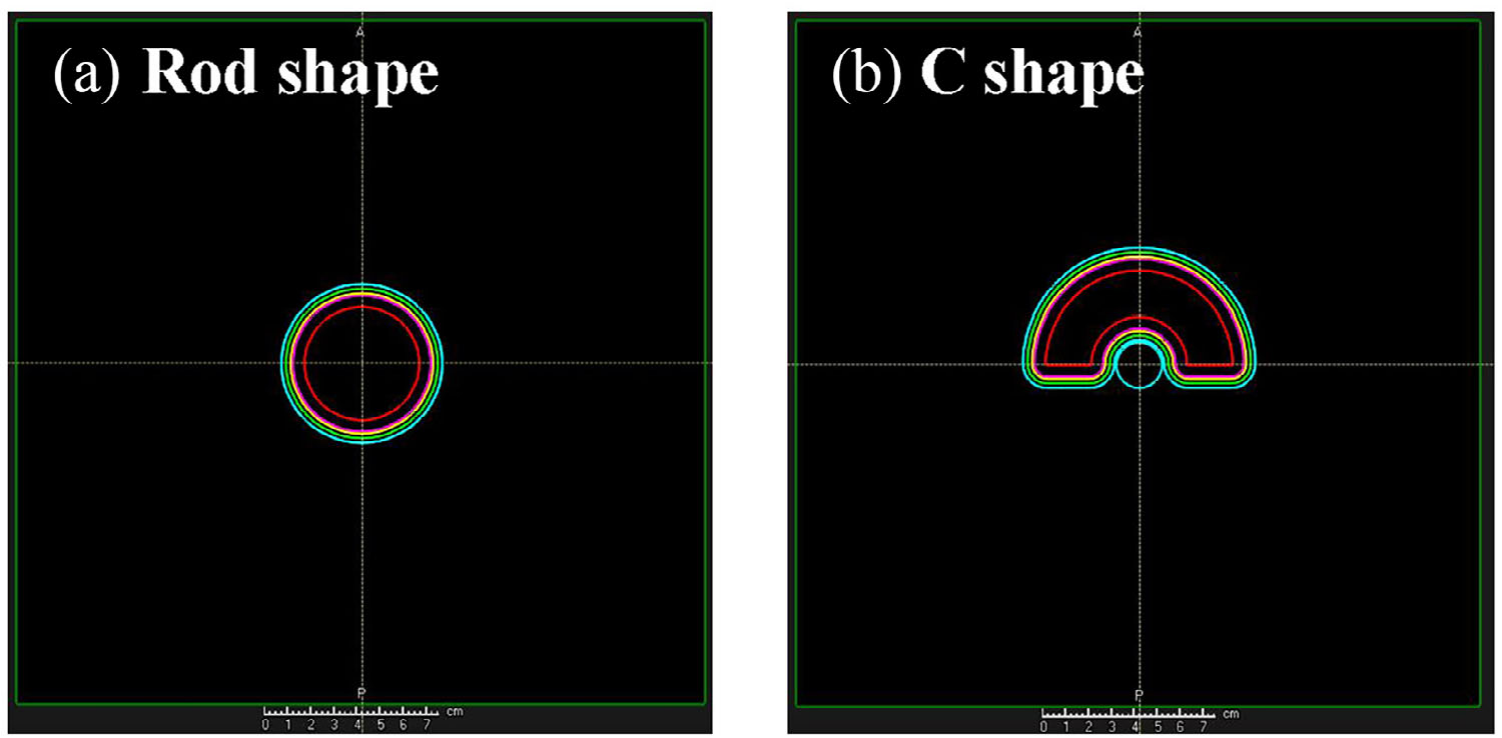
Phantom geometries used in this study. (A) Rod‐shaped target: a cylindrical GTV (diameter, 5 cm; red) with 5‐mm CTV expansion (yellow) and PTV margin (green). (B) C‐shaped target: a concave GTV (width, 2 cm; red) surrounding a central cylindrical OAR (diameter, 2 cm; cyan), with corresponding CTV (yellow) and PTV (green) expansions. Both geometries were created within a 30 × 30 × 30 cm^3^ water‐equivalent phantom.

### 2.2 Treatment planning and optimization

Treatment plans were generated using RayStation 12A with pencil beam scanning with a prescription of 50 Gy (RBE) in 10 fractions, a hypofractionated dose scheme representative of clinical protocols used for pelvic tumors, such as prostate cancer, normalized to 95% target volume coverage. In Section 1, two opposing lateral fields (90° and 270°) were used. For Section 2, seven beam configurations were evaluated (Figure S1), representing a range of clinically relevant approaches from simple two‐field setups to complex multi‐field arrangements: two lateral fields (2Lat: 90°/270°), two anterior oblique fields (2AO: 60°/300°), two posterior oblique fields (2PO: 120°/240°), anterior with right lateral fields (2AR: 0°/270°), anterior with two lateral fields (3ALR: 0°/90°/270°), four‐field box (4FB: 0°/90°/180°/270°), and four‐field oblique (4FO: 60°/120°/240°/300°).

Four optimization strategies were compared. In Section 1, the PTV‐based optimization was evaluated using three margin levels (PTV1mm, PTV3mm, and PTV5mm) to enable a comparison with robust optimization at the corresponding uncertainty magnitudes. PTV‐based optimization applied dose objectives (weight: 100) with a minimum dose of 50 Gy (RBE) and a maximum dose of 52.5 Gy (RBE) for both the PTV and CTV, with a uniform dose objective of 50 Gy (RBE) for the CTV; no constraints were applied to the target volumes. For the C‐shaped configuration, the OAR was optimized using a dose objective of 22 Gy (RBE) (weight: 50) combined with a hard constraint at a maximum dose of 24.5 Gy (RBE). These OAR dose limits were set at approximately 50% of the target prescription dose, consistent with the relative dose constraints applied in clinical hypofractionated carbon‐ion protocols.^34^, ^35^ Robust optimization applied identical objectives and weights directly to the CTV using the minimax (worst‐case) approach, where the optimizer minimized the worst‐case objective function value across all uncertainty scenarios. Three parameter sets were evaluated: (1) translation only (1, 3, or 5 mm in six directions); (2) density only (± 1%, ± 3%, or ± 5%); and (3) combined translation and density (± 1%/1 mm, ± 3%/3 mm, or ± 5%/5 mm). In Section 2, PTV3mm was used for PTV‐based optimization and ± 3%/3 mm for robust optimization.

### 2.3 Error simulation and evaluation

Systematic delivery errors were simulated to evaluate the robustness of each optimization strategy. Translation errors were applied in 14 directions: six primary directions corresponding to three degrees of freedom (superior–inferior, left–right, and anterior–posterior) and eight diagonal directions representing combined movements (e.g., left–inferior–anterior, right–superior–posterior). Three magnitudes were evaluated: 1, 3, and 5 mm. Density errors simulating range uncertainties were applied as positive and negative variations at ± 1%, ± 3%, and ± 5%. The combined error scenarios applied translation and density errors simultaneously, yielding 28 scenarios per magnitude level (14 translation directions × 2 density directions).

As the robust evaluation function in RayStation 12A does not provide LET distribution data, each error scenario was calculated using the “Compute Perturbed Dose” function. This function applies the same perturbation methodology as the robust evaluation, including isocenter shifts for translation errors and computed tomography density scaling for range errors, while enabling the extraction of both dose and LET values under identical error conditions.

### 2.4 Evaluation metrics

The LETd and RBE‐weighted dose distributions were calculated under nominal and perturbed conditions. The following metrics were extracted for the GTV, CTV, and OAR: LETd98% (near‐minimum LETd at the 98th percentile of the volume), LETd mean (LETd across the volume), LETd2% (near‐maximum LETd at the 2nd percentile), and the corresponding clinical dose metrics (RBE‐weighted dose: D98%, mean dose, and D2%). The percentage change (%Δ) was calculated as the relative difference between perturbed and nominal values for each metric. To quantify LET robustness across uncertainty levels, two summary measures were calculated: the AVG IQR, representing overall variability across uncertainty magnitudes, and the S1→5, describing the rate of increase in LET variability from the smallest to the largest uncertainty magnitude. The S1→5 was calculated using the following formula:

$$S_{1\to 5}\;=\;\left({\text{IQR}}_{5}-{\text{IQR}}_{1}\right)/4$$

where IQR_5_ represents the IQR at the largest uncertainty level (5 mm or 5% or 5%/5 mm) and IQR_1_ represents the IQR at the smallest uncertainty level (1 mm or 1% or 1%/1 mm). The denominator of 4 corresponds to the interval between uncertainty levels (5−1 = 4 units).

The LETd metrics were derived from voxel‐based distributions within each structure, analogous to a cumulative LET–volume histogram. The IQR was selected to quantify the variability owing to the non‐normal distribution of the LET metrics under uncertainty conditions, providing a robust measure of dispersion. The S1→5 was introduced to characterize the rate of change in variability across increasing uncertainty magnitudes, enabling comparative assessment of robustness beyond single‐scenario analysis.

The AVG.IQR and sensitivity slope describe the spread of outcomes across the uncertainty set but do not indicate how far the least favorable scenario departs from the planned distribution. To quantify this, the nominal‐to‐worst‐case deviation was computed for each plan from the same scenario set. For each LETd metric, the most negative and most positive deviations from the nominal value were identified across all evaluated scenarios and expressed as percentages of the nominal value. The same quantity was computed for the RBE‐weighted dose. The most negative deviation of the target LETd98% and of the target D98% were taken as the primary indicators of underdosing risk in terms of LET and of dose, respectively, since each represents the largest reduction in near‐minimum coverage that any single scenario would produce.

### 2.5 Statistical analyses

All the statistical analyses were performed using SPSS version 30.0.0.0 (IBM Corporation, Armonk, NY, USA). The Shapiro–Wilk test was used to check whether the data followed a normal distribution. The results showed a significant deviation from normality (*p* < 0.05) for all the measured variables. Therefore, nonparametric statistical methods were used for all the analyses.

Results are reported as medians with IQRs or medians [Q1–Q3]. In Section 1, LET robustness was evaluated using descriptive statistics (AVG.IQR and S1→5). In Section 2, the Wilcoxon signed‐rank test was used to compare the PTV‐based and robust optimization strategies for each beam configuration, looking at them in pairs. To account for multiple comparisons, a Bonferroni correction was applied, which set a new significance level of *p* < 0.007 (0.05 divided by the seven beam configurations).

A comprehensive ranking system was developed to identify the optimal beam configuration for minimizing LETd variations under delivery errors. For each LETd metric (LETd98%, LETd mean, LETd2%), the beam configurations were ranked from 1 (best) to 7 (worst) based on their performance, and the total score was calculated as the sum of the individual ranks across all metrics. Two ranking criteria were evaluated: (i) a minimal‐change criterion that prioritized the smallest absolute LET variations and (ii) a biologically favorable criterion that favored increases in target LETd98% and LETd mean. LETd2% was excluded from the target volume ranking under this criterion because no preferred direction of change can be assigned to it: an increase may reflect either enhanced biological effectiveness within the target or an excessive high‐LET region, and present evidence does not establish which interpretation should govern. For OARs, reductions in all LETd metrics were considered desirable. Notably, ranking outcomes may vary depending on the relative weighting assigned to the target versus OAR metrics. However, in the present study, equal weighting was applied to both structures to provide a balanced evaluation.

### 2.6 Spot weight distribution analysis

To investigate the mechanistic basis of the differences in LET robustness between optimization strategies, spot‐level data were extracted from all treatment plans using the RayStation scripting interface (CPython 3.11). For each plan, the nominal energy, lateral position, and relative weight of every scanning spot were exported. Spot weights are reported as relative values normalized such that the total weight of each field equals unity; absolute monitor units were not available through this interface, and therefore only the relative distribution of weights within a field can be compared across plans.

Because the range of energy layers differs between beam directions and between target geometries, the energy of each spot was normalized within its own field as (E − Emin)/(Emax − Emin), yielding a relative depth coordinate ranging from 0 (most proximal layer) to 1 (most distal layer). Each field was then divided into proximal, mid, and distal thirds along this coordinate, and the fraction of total field weight deposited in each third was computed. Three metrics were derived per field: the number of discrete energy layers, the coefficient of variation (CV) of spot weights, and the weight fraction in each depth third. Field‐level values were averaged across all fields within a plan.

For Section 1, plans were grouped by the type of robustness parameter applied: PTV‐based, translation‐only robust, density‐only robust, and combined translation–density robust, with each group comprising the 1, 3, and 5 settings. For Section 2, differences between PTV‐based and robust optimization were compared across the seven beam configurations using the Wilcoxon signed‐rank test, and the relationship between the number of fields and the change in spot weight CV was assessed using Pearson correlation. Given the small number of configurations (*n* = 7), these analyses are considered exploratory.

## 3 RESULTS

### 3.1 LET robustness for organ at risk and target

In Section 1, two opposing lateral fields were used to assess the effect of the optimization strategies on the LET robustness. The robustness of the LET metrics (LETd98%, LETd mean, and LETd2%) under delivery uncertainty is summarized in Figure 2 and Table S1. The representative LETd distributions comparing the optimization strategies are presented in Figure S‐2. The results are presented as medians with IQRs because statistical analysis using the Shapiro–Wilk test revealed a significant deviation from the normal distribution (*p* < 0.05).

**FIGURE 2:**
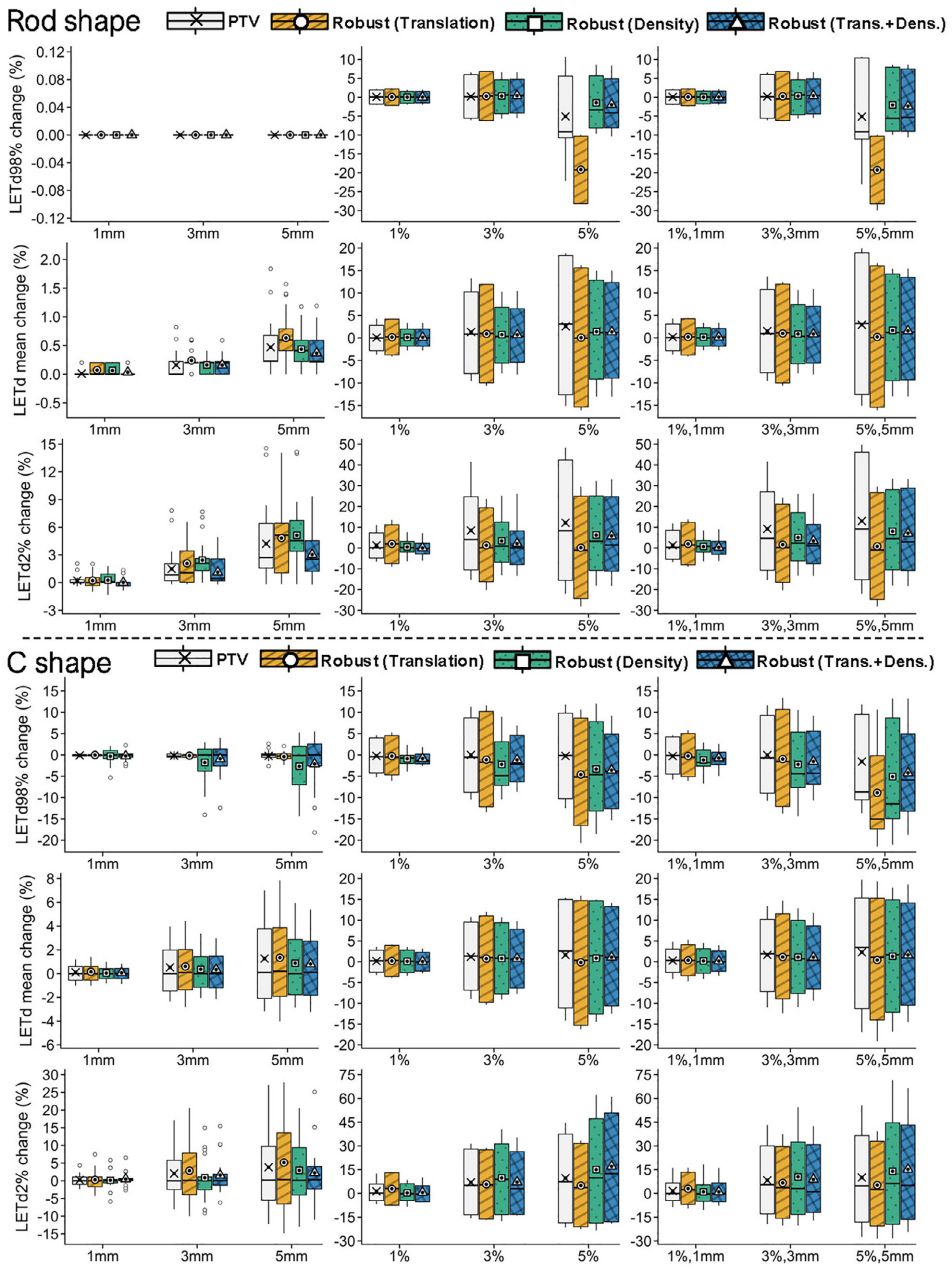
Distributions of percentage changes in LETd metrics under translation, density, and combined uncertainties in carbon‐ion therapy: Boxplots of percentage changes in LETd98%, LETd mean, and LETd2% for rod‐ and C‐shaped targets under translation, density, and combined translation–density uncertainties, comparing PTV‐based and robust optimization strategies.

The LET robustness was largely preserved under translation uncertainty for both the CTV and OARs. Rod‐shaped targets exhibited negligible variability in all metrics (AVG.IQR and S1→5 approaching zero). For C‐shaped targets, modest variability was introduced by translation uncertainty, particularly for LETd2%. For LETd2%, robust optimization that considered both translation and density effects produced the lowest AVG.IQR and S1→5.

Significant LETd variability with noticeable geometry‐dependent effects was caused by density uncertainty. LETd2% showed steep S1→5 and high AVG.IQR values for the C‐shaped targets. The OAR metrics showed trends similar to those of the CTV, but with significantly greater magnitudes. For both the CTV and OAR metrics, density‐aware robust optimization, specifically the combined translation–density strategy, achieved the lowest variability.

Combined translation‐density uncertainties represented the worst‐case scenario, with LETd2% showing the steepest S1→5 (Figure 2, bottom row; Table S1, “Combined” columns). Robust optimization incorporating density effects consistently achieved lower AVG.IQR values, confirming that density‐related uncertainties dominate LET variability and that density‐aware robust optimization enhances LET stability.

### 3.2 Different responses of LET and relative biological effectiveness‐weighted dose metrics

Figure 3 shows the median percentage changes in the LETd and RBE‐weighted dose metrics for the CTV under increasing uncertainty. The translation‐ and density‐only scenarios exhibited similar directional trends, but the combined scenario was shown as a typical case.

**FIGURE 3:**
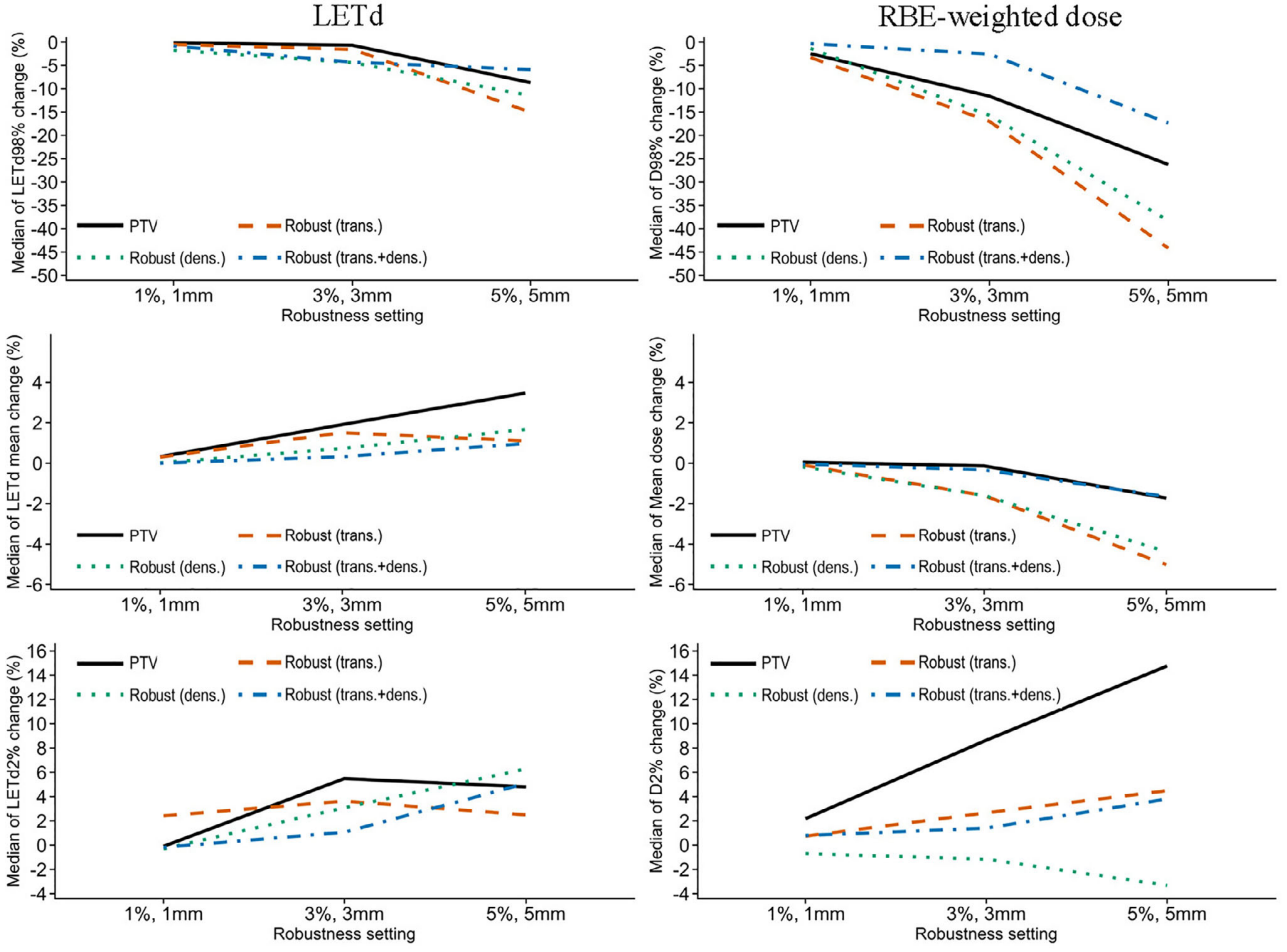
Median percentage changes of LET and RBE‐weighted dose metrics under increasing combined translation and density uncertainties for a C‐shaped target.

The RBE‐weighted dose coverage (D98%) was more sensitive than LETd98% and showed larger reductions when density uncertainties were present. The average dose metrics exhibited opposite trends. The median LETd mean increased because higher LET regions were sampled more often, whereas the median average RBE‐weighted dose decreased because the RBE‐weighted dose response was mostly due to physical dose loss. For both LET and RBE‐weighted doses, near‐maximum metrics (LETd2% and D2%) generally increased, which was consistent with tail amplification under range perturbations when the range changes.

These trends were more pronounced for OARs, as quantified in Table S1, which presents the AVG.IQR and S1→5 for each LETd metric under translation, density, and combined uncertainties. Notably, the OAR LETd2% exhibited the highest AVG.IQR values (up to 165% under density errors for the C‐shaped geometry), indicating its extreme sensitivity to range uncertainties. The RBE D98% had larger reductions, the median LETd mean increased, and the RBE D2% increases were more noticeable than those in the CTV. The largest reduction in the RBE‐weighted dose and the strongest tail amplification came from the PTV‐based optimization. In contrast, the combined robust optimization provided the most balanced response across all metrics.

The C‐shaped geometry was always more sensitive than the rod‐shaped geometry, which served as a stringent test to check the robustness of the differential LET and RBE‐weighted dose. These results indicate that the LET‐ and RBE‐weighted dose metrics react differently to uncertainty. This demonstrates the importance of evaluating the robustness of carbon‐ion therapy plans.

### 3.3 Nominal LET properties for various beam configurations

The impact of the beam configuration on the LET characteristics was evaluated in Section 2. The nominal LET metrics for each of the seven beam configurations are listed in Table S2, and the corresponding LETd distributions are shown in Figure S1. The nominal LETd98% values for the CTV were 29.1–44.1 keV/µm (PTV‐based) and 32.0–41.0 keV/µm (robust). The highest target LET levels were generated by multi‐field configurations, particularly the 4FB. Higher nominal LETd mean and LETd2% values were typically the outcomes of PTV‐based optimization, suggesting an increased high‐LET contribution at the baseline.

The OAR metrics varied more among the configurations. LETd2% showed strong sensitivity to the beam direction and optimization technique, with elevated values for the PTV‐based optimization in multiple configurations. In general, a more moderate baseline LET distribution was produced by robust optimization by reducing the nominal LETd mean and LETd2% compared with the PTV‐based optimization.

### 3.4 Beam direction's impact on LET robustness

Table S3 summarizes the beam direction‐dependent LET changes under delivery uncertainties. For statistical comparisons, the Wilcoxon signed‐rank test was used, and the median percentage changes with IQR were used to display the results.

The median LETd98% changes in the CTV were generally insignificant across beam configurations. The LETd mean exhibited greater sensitivity to the beam direction, and robust optimization reduced the dispersion compared with the PTV‐based optimization for most configurations. LETd2% demonstrated the strongest beam direction dependence, and the PTV‐based optimization consistently resulted in broader IQRs and larger increases (*p* < 0.001 for all configurations).

For the OAR LET metrics, the effects of beam direction were more pronounced. The large IQRs for LETd2% in the two‐beam and oblique configurations showed that the high‐LET regions were highly sensitive to delivery uncertainties. The magnitude and variability were consistently reduced by the robust optimization of the OAR LETd2% in all configurations (*p* < 0.001). These results demonstrate that the beam direction significantly affects LET robustness, with LETd2% showing the strongest dependence. Robust optimization systematically reduced the beam direction‐dependent variability for both the CTV and OARs.

### 3.5 Beam configurations ranking analysis

For both the PTV‐based and robust optimizations, the two lateral field (2Lat) configurations consistently obtained the highest ranking, achieving the lowest total score of 26 under the PTV‐based optimization and 27 under the robust optimization (Tables S3, S4, and S5) under the minimal‐change criterion, exhibiting superior physical stability with minimal LET variations. According to this criterion, multi‐field configurations (4FB and 4FO) were ranked second under PTV‐based optimization, whereas 2AO and 2PO ranked second and third, respectively, under robust optimization. Complete ranking data, including individual metric scores for CTV, GTV, and OAR, are provided in Tables S4 and S5.

Conversely, the multi‐field configurations performed better under biologically favorable conditions. Under the PTV‐based optimization, two anterior with right lateral fields (2AR) and 4FB shared the first rank, followed by 4FO and three‐field (3ALR) configurations tied at the third rank. Under robust optimization, 4FO was ranked first, demonstrating the best overall biologically favorable LET distribution, followed by 2Lat (rank 2) and 2AO (rank 3).

These findings indicate that biologically advantageous behaviors do not always correlate with physical robustness. The 2Lat configuration performed moderately under the biologically favorable criterion with robust optimization (rank 2) while achieving the highest minimal‐change ranking. Notably, 4FO with robust optimization ranked first under the biologically favorable criterion but only sixth (tied with 3ALR) under the minimal‐change criterion, illustrating that no single configuration achieved both biological advantage and physical stability. According to these results, multi‐field configurations, especially 4FO with robust optimization, are favored when biological effectiveness is the main goal, whereas 2Lat should be used when physical LET stability is the top priority.

### 3.6 Spot weight distribution

For the cylindrical target, the type of robustness parameter determined whether the spot pattern differed from PTV‐based optimization (Table 1). Density‐based robust optimization, applied either alone or in combination with translation, produced a more homogeneous spot weight distribution than PTV‐based optimization (CV 0.917 and 0.915 vs. 1.093) and used a greater number of energy layers (38.0 vs. 36.3). Weight was correspondingly redistributed away from the distal region, with the distal‐third weight fraction decreasing from 60.8% for PTV‐based optimization to 56.4% and 57.4% for density‐only and combined robust optimization, respectively, and the proximal‐third fraction increasing from 7.8% to approximately 12%.

**TABLE 1:** Spot weight distribution by optimization strategy (Section 1).

Target	Optimization strategy	Energy layers	Spot weight CV	Proximal third (%)	Mid third (%)	Distal third (%)
Cylindrical	PTV‐based	36.3 ± 1.2	1.093 ± 0.013	7.8 ± 0.7	31.4 ± 1.7	60.8 ± 1.1
	Robust: translation only	33.0 ± 0.0	1.074 ± 0.007	8.9 ± 0.5	27.4 ± 0.7	63.7 ± 1.3
	Robust: density only	38.0 ± 3.0	0.917 ± 0.016	12.0 ± 1.3	31.6 ± 1.5	56.4 ± 0.2
	Robust: translation + density	38.0 ± 3.0	0.915 ± 0.021	11.8 ± 1.1	30.8 ± 2.0	57.4 ± 0.8
C‐shaped	PTV‐based	52.0 ± 2.0	1.094 ± 0.052	30.4 ± 0.3	30.6 ± 1.6	39.0 ± 1.4
	Robust: translation only	49.0 ± 0.0	1.060 ± 0.085	29.8 ± 0.9	28.2 ± 1.0	42.0 ± 1.9
	Robust: density only	53.7 ± 2.5	1.199 ± 0.072	27.1 ± 1.5	33.9 ± 2.1	38.9 ± 0.8
	Robust: translation + density	54.0 ± 3.0	1.219 ± 0.074	27.6 ± 1.4	32.2 ± 1.1	40.2 ± 2.5

*Note*: Values are mean ± SD across the three uncertainty settings (1, 3, and 5; *n* = 3 per group). Spot weights are relative values normalized to unity per field. Depth thirds are defined by energy normalized within each field, where 0 is the most proximal and 1 the most distal layer.

Abbreviation: CV, coefficient of variation.

Translation‐only robust optimization showed the opposite behavior. It used fewer energy layers than PTV‐based optimization (33.0 vs. 36.3), retained a comparable spot weight CV (1.074 vs. 1.093), and increased rather than decreased the distal‐third weight fraction (63.7% vs. 60.8%).

For the C‐shaped target, these differences were not reproduced. Density‐based robust optimization yielded a higher spot weight CV than PTV‐based optimization (1.199 and 1.219 for density‐only and combined, vs. 1.094), and the distal‐third weight fractions were similar across all four strategies (38.9%–42.0%). The number of energy layers followed the same ordering as for the cylindrical target, with density‐based strategies using more layers (53.7 and 54.0) than PTV‐based optimization (52.0) and translation‐only robust optimization using fewer (49.0).

Across the seven beam configurations evaluated in Section 2 (Table S6), robust optimization consistently used more energy layers than PTV‐based optimization, with increases of 1.5 to 2.0 layers per field in every configuration (Wilcoxon signed‐rank, *p* = 0.016); this corresponds to a relative increase of approximately 4%. The change in spot weight CV between optimization strategies was not consistent in direction across configurations (*p* = 0.375) but was inversely correlated with the number of fields (*r* = −0.864, *p* = 0.012): CV increased with robust optimization in the two‐field configurations 2Lat, 2AR, and 2PO, and decreased in the multi‐field configurations 3ALR, 4FB, and 4FO. No consistent redistribution of weight between depth regions was observed across configurations (*p* = 0.469).

### 3.7 Nominal‐to‐worst‐case deviation

For the rod‐shaped target, the optimization strategy determined the magnitude of the worst‐case reduction in target LETd98% (Table S7). PTV‐based optimization produced a worst‐case reduction of 14.3%. Robust optimization incorporating density uncertainty, either alone or in combination with translation, reduced this to 8.4% and 8.8%, respectively, whereas translation‐only robust optimization increased it to 29.1%. The same ordering was observed for LETd mean, for which the worst‐case reduction fell from 13.3% under PTV‐based optimization to 10.4% and 10.1% under density‐based and combined robust optimization, and rose to 15.5% under translation‐only robust optimization.

For the C‐shaped target, this ordering was not reproduced. All robust strategies produced worst‐case LETd98% reductions larger than that of PTV‐based optimization (17.0% and 16.7% for density‐based and combined robust optimization, and 20.8% for translation‐only robust optimization, vs. 11.7%). The worst‐case reductions in LETd mean were comparable across strategies, ranging from 13.6% to 18.2%.

Across the seven beam configurations evaluated in Section 2, all of which used the C‐shaped geometry, robust optimization produced a larger worst‐case reduction in target LETd98% than PTV‐based optimization in six of the seven configurations (Table S8), although the difference was not statistically significant (Wilcoxon signed‐rank, *p* = 0.078) and exceeded two percentage points only for 2PO (27.1% vs. 18.3%). Worst‐case reductions in LETd mean were smaller under robust optimization in five of the seven configurations.

The corresponding analysis of the RBE‐weighted dose gave the opposite pattern (Tables S7 and S8). For the rod‐shaped target, the worst‐case reduction in target D98% was 32.8% under PTV‐based optimization and 23.5% under combined robust optimization, while translation‐only robust optimization increased it to 51.2%. For the C‐shaped target the corresponding values were 77.6%, 65.8%, and 84.1%. Across the seven beam configurations, robust optimization reduced the worst‐case loss of D98% in every configuration (Wilcoxon signed‐rank, *p* = 0.016), with reductions ranging from 7.5 percentage points for 2Lat to 23.7 percentage points for 2AR; the worst‐case reduction in mean dose was likewise smaller under robust optimization in all seven configurations (*p* = 0.016).

The worst‐case values for both quantities arose from the largest uncertainty scenario evaluated (5%/5 mm), which is beyond the magnitude for which the robust plans in Section 2 were optimized (3%/3 mm) and larger than the range uncertainty of approximately 3.5% typically assumed clinically. For the C‐shaped target, the worst‐case dose scenario was a posterior shift combined with a density increase; the mean dose in that scenario fell by only 4.6%, indicating a localized cold region within the concavity rather than a uniform loss of coverage, which the near‐minimum metric D98% amplifies. The worst‐case LET scenario was a density decrease, that is, the opposite direction of density error, consistent with the divergent responses of LET and dose reported in Section 3.2.

## 4 DISCUSSION

This study systematically evaluated the robustness of the LET metrics. The main findings are as follows: (1) density‐related uncertainties dominate LET variability, especially LETd2%; (2) robust optimization with combined translation‐density parameters provides superior LET stability; (3) beam configuration selection depends on optimization priorities, with multi‐field configurations offering biologically favorable LET distributions whereas two lateral fields provide superior physical stability; and (4) spot weight distribution analysis indicates that only robustness scenarios including density uncertainty redistribute weight away from distal energy layers, providing a mechanistic account of the observed differences in LET stability. This study differs from previous studies in that it provides the first systematic comparison of LET robustness across seven beam configurations, establishes a dual‐criteria ranking methodology, and characterizes the divergent behavior of LET and clinical dose metrics under identical uncertainty scenarios.

### 4.1 LET robustness characteristics under delivery uncertainties

The “LET trilemma”—an intrinsic conflict among robust target coverage, uniform dose, and high LETd mean in carbon therapy—described by Fredriksson et al.^24^ is consistent with the sensitivity of LET distributions to delivery uncertainties observed in this study. The present results confirm that high‐LET regions close to the distal edge are preferentially affected by range uncertainties, with LETd2% showing the highest sensitivity among all metrics. Although Parrella et al.^25^ showed that acceptable robustness could still be achieved with LET optimization through careful planning strategies, Schafasand et al.^11^ similarly reported that increasing the LETd mean compromised the plan robustness in sacral chordomas.

Yagi et al.^36^ found that robust radiobiological optimization reduced the impact of range and position uncertainties on dose distribution by up to 19%. Although this value pertains to dose robustness rather than LET specifically, it supports the general efficacy of robust optimization approaches in mitigating delivery uncertainties. In terms of beam configuration effects, Shin et al.^29^ found that oblique beam configurations were more robust than parallel opposed beams in the treatment of prostate cancer, with parallel configurations exhibiting a 1.5‐fold higher LET CTV D95% standard deviation. Our results suggest that the effects of the beam configuration on LET robustness depend on the evaluation criterion, with multi‐field arrangements offering biologically favorable LET distributions and two lateral fields offering superior physical stability, a distinction not previously characterized in the literature.

Spot weight distribution analysis provides a mechanistic explanation for these differences between optimization strategies (Table 1). Each energy layer corresponds to a Bragg peak at a specific depth, and the highest LET values occur at the distal edge of each peak, a position that shifts when the actual range differs from the planned range. When weight is concentrated in relatively few distal layers, a range shift displaces these high‐LET regions substantially, producing large changes in the LET distribution. Distributing weight across a greater number of energy layers and shifting it towards more proximal depths increases the overlap between adjacent Bragg peaks, so that a range shift produces a smaller net change in the resulting LET distribution. For the cylindrical target, only robust optimization scenarios that explicitly included density uncertainty altered the spot pattern in this direction, using more energy layers (38.0 vs. 36.3), producing more homogeneous spot weights (coefficient of variation 0.915–0.917 vs. 1.093), and reducing the distal‐third weight fraction from 60.8% to 56.4–57.4%. Translation‐only robust optimization behaved in the opposite manner, using fewer energy layers (33.0) and increasing the distal‐third weight fraction to 63.7%. This asymmetry parallels the finding that density uncertainty, rather than setup uncertainty, dominated LET variability.

That this mechanism was evident for the cylindrical target but not for the C‐shaped target merits comment. In the C‐shaped geometry, the concave target surrounding an organ at risk imposes strong constraints on achievable spot patterns, requiring steep dose gradients that are produced by highly heterogeneous spot weights regardless of the optimization strategy. The spot weight coefficient of variation was correspondingly high for all four strategies (1.060–1.219), and the additional heterogeneity introduced by density‐based robust optimization exceeded any homogenizing effect. This suggests that the mechanism by which robust optimization improves LET stability operates most clearly when target geometry does not already dictate the spot pattern, and that its contribution may be reduced in anatomically constrained clinical scenarios.

The nominal‐to‐worst‐case deviation provides an independent test of the mechanism inferred from the spot weight distributions, and the two analyses agree. For the rod‐shaped target, the strategies that redistributed weight towards proximal energy layers, namely those incorporating density uncertainty, were also those that approximately halved the worst‐case reduction in target LETd98%, while translation‐only robust optimization, which increased reliance on distal layers, roughly doubled it. That two measures derived from different quantities, one from the spot pattern and one from the LET distribution itself, order the four strategies identically supports the interpretation that reliance on distal energy layers governs the sensitivity of LET to range uncertainty.

For the C‐shaped target the correspondence also holds, in that neither analysis showed an advantage for robust optimization. Where the concave geometry constrains the achievable spot pattern, robust optimization did not produce the proximal redistribution seen in the rod‐shaped target, and correspondingly did not reduce the worst‐case loss of LETd98%. The same applies to the beam configuration comparison in Section 2, which used the C‐shaped geometry throughout. These results distinguish two aspects of robustness that are often treated together: robust optimization narrowed the central spread of LET outcomes, as measured by AVG.IQR, in both geometries, but reduced the worst‐case deviation only in the unconstrained geometry.

The contrast between the dose and LET results reinforces the central observation of this study. Robust optimization reduced the worst‐case loss of target D98% in every beam configuration and in both target geometries, which is the behavior it is designed to produce, since the minimax formulation acts on the dose objective. Over the same scenarios it did not reduce, and in the constrained geometry slightly increased, the worst‐case loss of target LETd98%. A plan selected on the basis of its dose robustness therefore carries no guarantee of comparable LET robustness, and the two must be evaluated separately. That the worst case for dose arose from a density increase while the worst case for LET arose from a density decrease makes the point concrete: no single scenario represents the least favorable outcome for both quantities.

A key finding of this study was the non‐parallel evolution of LET‐ and clinical dose (RBE‐weighted dose)‐based metrics under uncertainty. Density‐related uncertainties caused LETd to increase, whereas the mean clinical dose decreased, indicating LET redistribution toward distal regions along with a decrease in the absorbed dose. This divergence is in line with the findings of Inaniwa et al.,^14^ who stressed that explicit consideration of both quantities is necessary for dose and LET optimization. Similarly, Masuda et al.^37^ found that in multi‐ion therapy, range undershoot increased the LETd mean, whereas overshoot decreased it. Instead of depending only on the clinical dose (RBE‐weighted dose) metrics, these results encourage explicit LET evaluation.

### 4.2 Implications for LET‐based treatment strategies

The observed LET sensitivity has implications for advanced treatment strategies, such as LET painting. Sokol and Durante^9^ noted that high‐LET regions remained clinically underutilized in hypoxic tumors. Bassler et al.^38^ and Kohno et al.^39^ demonstrated the potential of LET painting in optimizing biological efficacy. Our results suggest that robust optimization is necessary to maintain the intended LET distributions, as LETd2% sensitivity to density uncertainties may compromise the biological efficacy of LET‐painted plans.

Further context is provided through a comparison with LET studies on proton therapy. High LET can be redistributed from OARs to tumors with little impact on robustness, according to Liu et al.’s^40^ demonstration of LET‐guided robust optimization for head and neck proton therapy. Because heavier ion beams have higher LET gradients, the carbon‐ion results presented here show more noticeable LET variations, highlighting the significance of LET‐aware robust optimization in carbon‐ion therapy.

### 4.3 Clinical implications

Based on these findings, we suggest the following clinical recommendations. First, LET robustness should be evaluated alongside dose robustness in radioresistant or hypoxic tumors. Second, beam configuration selection should reflect clinical priorities: 4FO with robust optimization for biological effectiveness or 2Lat for physical stability. Robust optimization is essential for maintaining the LET stability in facilities with limited beam ports, where two‐beam configurations are inevitable.

This second recommendation requires qualification. The spot weight distribution analysis showed that configurations differ markedly in how uniformly their constituent fields contribute across depth. In configurations where all fields traverse comparable depths, such as 2Lat, 2AO, and 2PO, the weighted mean depth differed between fields by a standard deviation of less than 0.01 in normalized units. In asymmetric configurations, including 2AR, 3ALR, 4FB, and 4FO, the corresponding values ranged from 0.073 to 0.142, indicating that individual fields contribute dose and LET at substantially different depths. Plan‐level averages therefore conceal considerable variation between fields, and the ranking of a configuration reflects the particular field arrangement by which it was realized in this phantom rather than an intrinsic property of the beam angles themselves. The performance of 2Lat also depends on unobstructed lateral beam access and on setup and motion characteristics that a phantom cannot represent; at anatomical sites where lateral entry traverses variable anatomy or critical structures, its advantage in physical stability may not be retained. Beam configuration should therefore be selected case by case, with the rankings reported here used to indicate the direction of the trade‐off rather than to prescribe specific angles.

Third, the treatment planning workflows should include explicit LET‐based evaluations. If a clinical LET threshold is applied (e.g., CTV minimum LETd ≥ 42.2 keV/µm as suggested by Sakamoto et al.^13^ for pancreatic cancer), our results indicate that certain beam configurations and uncertainty scenarios may fail to achieve this criterion. Specifically, two‐field configurations under density perturbations showed reduced LETd values, which could potentially compromise biological effectiveness in clinical scenarios. Supporting this concern, Yoshimatsu et al.^40^ recently demonstrated that interfractional variations in bowel gas could cause distal shifts in LETd distributions during daily CIRT sessions, resulting in reduced LETd coverage of target volumes. This is particularly important for hypoxic tumors.^9^, ^41^ The ranking analysis showed that 4FO with robust optimization performed best under the biologically favorable criterion, whereas 2Lat ranked the highest under the minimal‐change criterion.

### 4.4 Limitations and future work

This study has some limitations. The analysis used simplified phantom geometries rather than patient‐specific anatomy, and intrafractional anatomical variations, which affect LET distributions,^40^ were not considered. LET‐based objectives were not included in the optimization. The uncertainty magnitudes (1–5 mm translation, ± 1–5% density) were selected to systematically investigate step‐wise impacts rather than replicate the clinically standard ± 3.5% range uncertainty.^18^, ^42^ Only the modified MKM with HSG parameters was assessed.^31^, ^32^

Further limitations apply to the spot weight distribution analysis. Spot weights were available only as relative values normalized to unity within each field rather than as absolute monitor units, so the shape of the weight distribution can be compared between plans but the absolute intensity delivered by a given field cannot. Dose‐averaged LET was computed only for the composite dose distribution, as the treatment planning system does not provide per‐field LET; the spot weight metrics are therefore an indirect surrogate for the per‐field mechanisms underlying LET robustness, and the mechanistic explanation proposed above remains inferential, since spot patterns were observed to differ in the predicted direction but were not manipulated independently of the optimization strategy. The depth coordinate was normalized within each field, so proximal, mid, and distal thirds describe the internal structure of each field relative to its own energy range and do not correspond to absolute depths within the phantom.

The comparison across beam configurations comprised seven configurations, each evaluated with a single pair of optimization strategies. The statistical power of paired tests at this sample size is low, and the absence of a significant difference should not be taken as evidence that none exists; the inverse correlation between the number of fields and the change in spot weight coefficient of variation was derived from seven data points and should be regarded as exploratory. All configurations in this comparison used the C‐shaped geometry, in which the relationship between optimization strategy and spot weight homogeneity differed from that observed for the cylindrical target, so these observations apply to a target constrained by an adjacent organ at risk and cannot be assumed to hold for less constrained geometries. An in silico study using patient datasets should be conducted as the next step, to establish whether these phantom‐based findings extrapolate to clinical anatomy in which tissue heterogeneity, organ motion, and site‐specific beam access are present. Future work should also incorporate 4D treatment planning and integrate LET‐based robustness objectives into clinical planning systems.

## 5 CONCLUSION

This phantom‐based study evaluated the robustness of LET metrics in carbon‐ion therapy by comparing optimization techniques and beam configurations. Density‐related uncertainties affected LET distributions more than translation errors, with LETd2% being the most sensitive metric. The LET and clinical dose (RBE‐weighted dose) metrics showed divergent responses under uncertainty, highlighting the need for explicit LET evaluation. Robust optimization incorporating translation and density parameters achieved greater LET stability than PTV‐based optimization, and spot weight distribution analysis indicated that this arises from a redistribution of weight away from distal energy layers; in the unconstrained rod‐shaped geometry this was accompanied by an approximate halving of the worst‐case reduction in target LETd98%, whereas in the organ‐at‐risk‐constrained C‐shaped geometry no such reduction was observed. Under the criteria applied here, two lateral fields (2Lat) provided the greatest physical LET stability, whereas 4FO with robust optimization offered biologically favorable LET distributions with reduced OAR exposure; because these rankings reflect idealized phantom geometries and the specific field arrangements used, they indicate the direction of the trade‐off rather than prescribing beam angles for clinical use. These findings support the explicit consideration of LET robustness alongside dose robustness in carbon‐ion treatment planning, and warrant validation in in‐silico patient studies.

## AUTHOR CONTRIBUTIONS

**Wisawa Phongprapun**: Conceptualization, Methodology, Formal analysis, Investigation, Data curation, Writing – original draft, Visualization. **Mutsumi Tashiro**: Conceptualization, Writing – review & editing, Supervision.

## CONFLICT OF INTEREST STATEMENT

The authors declare no conflicts of interest.

## ETHICS STATEMENT

This study involved no human participants or patient data. All analyses were performed using computational phantoms; therefore, institutional review board approval was not required.

## Supporting information




Supporting Information

## Data Availability

The data that support the findings of this study are available from the corresponding author upon reasonable request.
