# Supplementary material for: Impact of robust optimization and beam configuration on the distribution stability of LET under delivery uncertainties in carbon‐ion therapy
Source: J Appl Clin Med Phys. 2026 Oct 27;27(11):e70878. doi: 10.1002/acm2.70878 (PMC13714341; doi:10.1002/acm2.70878)
Supplement: Supplementary file 1 [file ACM2-27-e70878-s001.docx]

**Supplementary Files**

Supplementary Figure 1. Dose-averaged LET (LETd) distributions for C-shaped target geometry across seven beam configurations. Panels 1–7 show PTV-based optimization (3 mm margin); panels 8–14 show robust optimization (±3%/3 mm). Beam configurations: (1, 8) two lateral fields (2Lat); (2, 9) anterior with right lateral (2AR); (3, 10) two anterior oblique (2AO); (4, 11) two posterior oblique (2PO); (5, 12) anterior with two lateral (3ALR); (6, 13) four-field box (4FB); and (7, 14) four-field oblique (4FO). Yellow arrows indicate beam directions. Color scale represents LETd in keV/μm.

Supplementary Figure 2. Dose-averaged LET (LETd) distributions for rod-shaped (1–4) and C-shaped (A–D) target geometries under different optimization strategies using two opposing lateral beams. (1, A) PTV-based optimization with 5 mm margin; (2, B) robust optimization with 5 mm translation uncertainty; (3, C) robust optimization with ±5% density uncertainty; and (4, D) robust optimization with combined 5 mm translation and ±5% density uncertainties. Yellow contour indicates the CTV; cyan arrows indicate beam directions. Color scale represents LETd in keV/μm.

Supplementary Table 1. Quantitative summary of LET robustness for target and OAR in carbon-ion therapy: Averaged interquartile ranges (AVG.IQR) and sensitivity slopes (S1→5) of LETd98%, LETd mean, and LETd2% for the clinical target volume (CTV) and organs at risk (OARs) under translation, density, and combined translation–density uncertainties for rod-s and C-shaped geometries.

| Contour | Error type | Shape | Parameter | LETd98% | | | | LETd mean | | | | LETd2% | | | |
| --- | --- | --- | --- | --- | --- | --- | --- | --- | --- | --- | --- | --- | --- | --- | --- |
|  |  |  |  | PTV | Robust translation | Robust density | Robust Trans&Dens | PTV | Robust translation | Robust density | Robust Trans&Dens | PTV | Robust translation | Robust density | Robust Trans&Dens |
| CTV | Translation | Rod | AVG.IQR | 0.00 | 0.00 | 0.00 | 0.00 | 0.23 | 0.20 | 0.26 | 0.20 | 2.33 | 3.22 | 1.85 | 2.03 |
|  |  |  | S1->5 | 0.00 | 0.00 | 0.00 | 0.00 | 0.11 | 0.04 | 0.04 | 0.10 | 1.13 | 1.12 | 0.60 | 0.74 |
|  |  | C-shape | AVG.IQR | 0.64 | 0.68 | 5.37 | 3.33 | 3.50 | 3.44 | 2.69 | 2.69 | 8.52 | 11.54 | 6.02 | 3.38 |
|  |  |  | S1->5 | 0.14 | 0.23 | 1.76 | 1.08 | 1.18 | 1.15 | 0.95 | 0.93 | 3.27 | 4.31 | 3.11 | 1.43 |
|  | Density | Rod | AVG.IQR | 10.52 | 11.71 | 8.66 | 8.33 | 18.25 | 20.25 | 12.73 | 12.34 | 35.09 | 34.53 | 20.07 | 19.13 |
|  |  |  | S1->5 | 3.14 | 3.36 | 2.66 | 2.48 | 6.32 | 5.74 | 4.55 | 4.36 | 11.44 | 7.65 | 7.66 | 7.68 |
|  |  | C-shape | AVG.IQR | 15.24 | 18.92 | 11.05 | 10.24 | 15.94 | 19.42 | 16.64 | 14.20 | 35.94 | 38.92 | 40.39 | 39.56 |
|  |  |  | S1->5 | 2.96 | 4.00 | 4.76 | 3.80 | 5.20 | 5.62 | 5.45 | 4.83 | 11.39 | 8.04 | 13.83 | 14.67 |
|  | Trans&Dens | Rod | AVG.IQR | 12.30 | 11.75 | 9.87 | 9.64 | 18.65 | 20.51 | 13.67 | 13.14 | 37.68 | 36.48 | 22.50 | 21.62 |
|  |  |  | S1->5 | 4.43 | 3.37 | 3.38 | 3.32 | 6.40 | 5.85 | 4.90 | 4.72 | 11.88 | 7.74 | 8.30 | 8.33 |
|  |  | C-shape | AVG.IQR | 15.67 | 16.70 | 13.48 | 11.17 | 16.51 | 19.05 | 16.85 | 14.92 | 36.23 | 39.67 | 40.07 | 38.14 |
|  |  |  | S1->5 | 2.84 | 1.77 | 4.95 | 3.79 | 5.25 | 5.46 | 5.28 | 4.92 | 10.90 | 8.32 | 13.43 | 11.94 |
| OAR | Translation | C-shape | AVG.IQR | 0.28 | 0.39 | 0.00 | 0.41 | 10.73 | 8.97 | 6.60 | 6.34 | 8.78 | 35.27 | 14.69 | 4.00 |
|  |  |  | S1->5 | 0.21 | 0.19 | 0.00 | 0.00 | 3.47 | 2.95 | 2.24 | 2.39 | 2.87 | 11.96 | 5.33 | 1.26 |
|  | Density |  | AVG.IQR | 17.75 | 13.22 | 18.49 | 23.05 | 104.68 | 89.24 | 113.64 | 111.49 | 99.43 | 160.18 | 129.51 | 87.30 |
|  |  |  | S1->5 | 8.57 | 5.80 | 8.75 | 13.04 | 39.45 | 37.64 | 43.37 | 38.98 | 7.37 | 29.68 | 12.07 | 7.38 |
|  | Trans& Dens |  | AVG.IQR | 19.35 | 13.96 | 19.83 | 27.06 | 105.00 | 88.03 | 116.93 | 113.22 | 113.29 | 165.12 | 142.38 | 93.68 |
|  |  |  | S1->5 | 9.54 | 6.09 | 9.52 | 15.43 | 38.23 | 37.87 | 42.52 | 36.56 | 9.94 | 32.38 | 14.26 | 7.92 |

Supplementary Table 2. Nominal LET metrics for CTV, GTV, and OAR across different beam configurations under PTV-based and robust optimization in carbon-ion therapy

| Contour | Plan | LETd98% (keV/mm) | | LETd mean (keV/mm) | | LETd2% (keV/mm) | |
| --- | --- | --- | --- | --- | --- | --- | --- |
|  |  | Robust | PTV | Robust | PTV | Robust | PTV |
| CTV | 2AO | 32.8 | 29.1 | 45.5 | 45.9 | 68.5 | 77.0 |
|  | 2Lat | 34.8 | 35.0 | 47.4 | 48.7 | 79.5 | 83.0 |
|  | 2PO | 38.8 | 34.4 | 48.1 | 50.5 | 63.1 | 64.2 |
|  | 2AR | 32.0 | 36.3 | 44.6 | 48.7 | 61.5 | 67.6 |
|  | 3ALR | 38.4 | 41.3 | 48.3 | 49.5 | 68.9 | 65.9 |
|  | 4Box | 41.0 | 44.1 | 51.0 | 54.3 | 67.2 | 70.3 |
|  | 4Obl | 32.6 | 37.6 | 45.3 | 48.8 | 66.1 | 66.7 |
| GTV | 2AO | 32.8 | 29.2 | 44.0 | 43.8 | 63.3 | 63.8 |
|  | 2Lat | 35.3 | 35.1 | 44.8 | 45.8 | 67.5 | 70.1 |
|  | 2PO | 40.3 | 35.1 | 46.8 | 49.6 | 52.9 | 60.5 |
|  | 2AR | 31.7 | 36.1 | 42.9 | 47.1 | 54.9 | 59.9 |
|  | 3ALR | 37.9 | 40.9 | 46.4 | 48.2 | 56.2 | 54.1 |
|  | 4Box | 40.8 | 44.5 | 49.0 | 53.0 | 62.0 | 66.0 |
|  | 4Obl | 32.2 | 37.5 | 43.5 | 47.5 | 56.5 | 59.5 |
| OAR | 2AO | 23.5 | 23.9 | 31.9 | 32.2 | 90.0 | 101.7 |
|  | 2Lat | 24.1 | 23.7 | 31.9 | 29.8 | 107.0 | 72.6 |
|  | 2PO | 22.1 | 23.9 | 27.3 | 36.1 | 61.0 | 108.3 |
|  | 2AR | 24.4 | 24.8 | 34.2 | 37.6 | 59.8 | 72.8 |
|  | 3ALR | 31.8 | 29.4 | 42.3 | 42.2 | 71.9 | 81.1 |
|  | 4Box | 29.6 | 28.3 | 39.8 | 41.0 | 68.2 | 75.4 |
|  | 4Obl | 22.9 | 23.9 | 31.0 | 33.4 | 89.2 | 67.7 |

Supplementary Table 3. Beam-direction-dependent changes in LET metrics for CTV and OAR under delivery uncertainties: comparison between PTV-based and robust optimization

| Contour | Field | Median %Change in LETd98% (keV/µm)/IQR | | p-value  (p < 0.007) | Median %Change in LETd mean (keV/µm)/IQR | | p-value  (p < 0.007) | Median %Change in LETd2% (keV/µm)/IQR | | p-value  (p < 0.007) |
| --- | --- | --- | --- | --- | --- | --- | --- | --- | --- | --- |
|  |  | Robust | PTV |  | Robust | PTV |  | Robust | PTV |  |
| CTV | 2AO | 0.00/1.83 | 0.00/4.47 | 0.797 | 0.00/6.81 | 0.00/10.68 | 0.177 | 0.15/13.87 | 0.26/23.08 | **<0.001** |
|  | 2AR | 0.00/2.32 | 0.00/3.18 | **<0.001** | 0.00/5.99 | 0.00/8.33 | 0.071 | 0.13/12.89 | 3.01/23.58 | **<0.001** |
|  | 2Lat | 0.00/6.32 | 0.00/9.71 | 0.512 | 0.00/5.27 | 0.00/7.19 | **0.001** | 0.00/11.70 | 0.00/15.78 | **<0.001** |
|  | 2PO | 0.00/2.32 | 0.44/7.63 | **<0.001** | 0.00/2.91 | 0.00/3.56 | 0.043 | –0.08/13.75 | 1.48/8.68 | **<0.001** |
|  | 3ALR | –0.38/3.07 | 0.14/4.05 | 0.889 | 0.00/5.42 | 0.20/8.13 | 0.048 | 0.20/11.47 | 0.26/18.30 | **<0.001** |
|  | 4FB | 0.00/3.17 | 0.00/2.72 | **<0.001** | 0.00/5.49 | 0.00/4.60 | 0.135 | 0.22/11.83 | 0.14/11.24 | **<0.001** |
|  | 4FO | 1.23/1.53 | 0.00/0.80 | **<0.001** | 0.00/5.74 | 0.00/5.12 | **<0.001** | 0.00/13.99 | 0.22/25.00 | **<0.001** |
| OAR | 2AO | 0.00/4.26 | 0.00/4.18 | **0.002** | 0.00/43.81 | 1.86/30.98 | 0.095 | 0.00/72.53 | 1.52/74.95 | **<0.001** |
|  | 2AR | 0.34/2.36 | 1.90/5.32 | **<0.001** | 2.99/12.44 | 0.87/21.57 | 0.231 | 5.64/37.50 | 0.00/41.78 | **<0.001** |
|  | 2Lat | 0.00/4.46 | 0.00/4.64 | 0.284 | 0.00/41.69 | 0.00/33.81 | 0.188 | 0.00/75.05 | 0.00/97.80 | **<0.001** |
|  | 2PO | 0.45/1.36 | 0.84/6.69 | 0.107 | 0.37/17.49 | 0.28/23.55 | 0.194 | 0.16/109.84 | 2.95/66.81 | **<0.001** |
|  | 3ALR | 0.35/2.10 | 0.68/3.63 | 0.979 | 3.17/15.36 | 1.17/28.55 | 0.154 | 5.59/47.04 | 0.06/59.28 | **<0.001** |
|  | 4FB | 0.34/6.42 | 0.00/5.57 | 0.105 | 2.26/16.27 | 7.07/14.39 | 0.256 | 6.16/57.51 | 2.98/28.18 | **<0.001** |
|  | 4FO | 1.31/3.49 | 1.67/6.28 | 0.700 | 0.00/30.00 | 6.29/21.26 | 0.131 | 0.39/83.07 | 0.00/62.22 | **<0.001** |

Supplementary Table 4. Beam configuration performance under PTV-based optimization: median percentage changes in LETd metrics with interquartile range (IQR) for CTV, GTV, and OAR and corresponding score rankings under minimal-change and biologically favorable criteria.

|  | **Dose metrics** | **Median of LETd(keV/mm)/IQR** | | | | | | | | **Score ranking** | | | | | | | |
| --- | --- | --- | --- | --- | --- | --- | --- | --- | --- | --- | --- | --- | --- | --- | --- | --- | --- |
|  |  | 2AO | 2AR | 2Lat | 2PO | 3ALR | | 4FB | 4FO | Criteria | 2AO | 2AR | 2Lat | 2PO | 3ALR | 4FB | 4FO |
| CTV | LETd 98% | 0.00 /4.47 | 0.00 /3.18) | 0.00 /9.71 | –0.44 /7.63 | –0.14 /4.05 | | 0.00 /2.72 | 0.00 /0.80 | Minimal change | 4 | 3 | 5 | 7 | 6 | 2 | 1 |
|  |  |  |  |  |  |  |  |  |  | Biologically favorable | 4 | 3 | 5 | 7 | 6 | 2 | 1 |
|  | LETd mean | 0.00 /10.68 | 0.00 /8.33 | 0.00 /7.19 | 0.00 /3.56 | 0.20 /8.13 | | 0.00 /4.60 | 0.00 /5.12 | Minimal change | 6 | 5 | 4 | 1 | 7 | 2 | 3 |
|  |  |  |  |  |  |  |  |  |  | Biologically favorable | 7 | 6 | 5 | 2 | 1 | 3 | 4 |
|  | LETd 2% | 0.26 /23.08 | 3.01 /23.58 | 0.00 /15.78 | 1.48 /8.68 | 0.26 /18.30 | | 0.14 /11.24 | 0.22 /25.00 | Minimal change | 5 | 7 | 1 | 6 | 4 | 2 | 3 |
|  |  |  |  |  |  |  |  |  |  | Biologically favorable |  |  |  |  |  |  |  |
| GTV | LETd 98% | 0.00 /4.11 | 0.00 /4.10 | 0.00 /10.75 | –0.28 /8.83 | 0.00 /10.76 | | 0.00 /1.80 | 0.00 /1.80 | Minimal change | 4 | 3 | 5 | 7 | 6 | 1 | 1 |
|  |  |  |  |  |  |  |  |  |  | Biologically favorable | 4 | 3 | 7 | 7 | 6 | 1 | 2 |
|  | LETd mean | 0.00 /7.93 | 0.00 /6.57 | 0.00 /5.46 | 0.00 /3.18 | 0.00 /6.02 | | 0.00 /2.64 | 0.00 /3.16 | Minimal change | 7 | 6 | 4 | 3 | 5 | 1 | 2 |
|  |  |  |  |  |  |  |  |  |  | Biologically favorable | 7 | 6 | 4 | 3 | 5 | 1 | 2 |
|  | LETd 2% | 0.26 /23.08 | 3.01 /23.58 | 0.00 /15.78 | 1.48 /8.68 | 0.26 /18.30 | | 0.14 /11.24 | 0.22 /25.00 | Minimal change | 5 | 7 | 1 | 6 | 4 | 2 | 3 |
|  |  |  |  |  |  |  |  |  |  | Biologically favorable |  |  |  |  |  |  |  |
| OAR | LETd 98% | 0.00 /4.18 | –1.90 /5.32 | 0.00 /4.64 | 0.84 /6.69 | –0.68 /3.63 | | 0.00 /5.57 | 1.67 /6.28 | Minimal change | 1 | 7 | 2 | 5 | 4 | 3 | 6 |
|  |  |  |  |  |  |  |  |  |  | Biologically favorable | 3 | 1 | 4 | 6 | 2 | 5 | 7 |
|  | LETd mean | 1.86 /30.98 | 0.87 /21.57 | 0.00 /33.81 | 0.28 /23.55 | 1.17 /28.55 | | 7.07 /14.39 | 6.29 /21.26 | Minimal change | 5 | 3 | 1 | 2 | 4 | 7 | 6 |
|  |  |  |  |  |  |  |  |  |  | Biologically favorable | 5 | 3 | 1 | 2 | 4 | 7 | 6 |
|  | LETd 2% | –1.52 /74.95 | 0.00 /41.78 | 0.00 /97.80 | –2.95 /66.81 | –0.06 /59.28 | | 2.98 /28.18 | 0.00 /62.22 | Minimal change | 5 | 1 | 3 | 6 | 4 | 7 | 2 |
|  |  |  |  |  |  |  |  |  |  | Biologically favorable | 2 | 4 | 6 | 1 | 3 | 7 | 5 |
|  | | | | | | | Total score | | | Minimal change | 42 | 42 | 26 | 43 | 44 | 27 | 27 |
|  |  |  |  |  |  |  |  |  |  | Biologically favorable | 32 | 26 | 30 | 28 | 27 | 26 | 27 |
|  |  |  |  |  |  |  | Rank | | | Minimal change | 4 | 4 | 1 | 6 | 7 | 2 | 2 |
|  |  |  |  |  |  |  |  |  |  | Biologically favorable | 7 | 1 | 6 | 5 | 3 | 1 | 3 |

Supplementary Table 5. Beam configuration performance under robust optimization: median percentage changes in LETd metrics with interquartile range (IQR) for CTV, GTV, and OAR and corresponding score rankings under minimal-change and biologically favorable criteria.

|  | **LETd** | **Median of LETd(keV/mm)/IQR** | | | | | | | | **Score ranking** | | | | | | | |
| --- | --- | --- | --- | --- | --- | --- | --- | --- | --- | --- | --- | --- | --- | --- | --- | --- | --- |
|  |  | 2AO | 2AR | 2Lat | 2PO | 3ALR | | 4FB | 4FO | Criteria | 2AO | 2AR | 2Lat | 2PO | 3ALR | 4FB | 4FO |
| CTV | D98% | 0.00 /1.83 | 0.00 /2.32 | 0.00 /6.32 | 0.00 /2.32 | –0.38 /3.07 | | 0.00 /3.17 | 1.23 /1.53 | Minimal change | 1 | 3 | 5 | 2 | 6 | 4 | 7 |
|  |  |  |  |  |  |  |  |  |  | Biologically favorable | 2 | 4 | 6 | 3 | 7 | 5 | 1 |
|  | Mean | 0.00 /6.81 | 0.00 /5.99 | 0.00 /5.27 | 0.00 /2.91 | 0.00 /5.42 | | 0.00 /5.49 | 0.00 /5.74 | Minimal change | 7 | 6 | 2 | 1 | 3 | 4 | 5 |
|  |  |  |  |  |  |  |  |  |  | Biologically favorable | 7 | 6 | 2 | 1 | 3 | 4 | 5 |
|  | D2% | 0.15 /13.87 | 0.13 /12.89 | 0.00 /11.70 | –0.08 /13.75 | 0.20 /11.47 | | 0.22 /11.83 | 0.00 /13.99 | Minimal change | 5 | 4 | 1 | 3 | 6 | 7 | 2 |
|  |  |  |  |  |  |  |  |  |  | Biologically favorable |  |  |  |  |  |  |  |
| GTV | D98% | 0.00 /2.13 | –0.26 /2.84 | 0.99 /3.68 | –0.62 /4.96 | 0.00 /3.02 | | 0.00 /3.13 | 1.55 /2.48 | Minimal change | 1 | 4 | 6 | 5 | 2 | 3 | 7 |
|  |  |  |  |  |  |  |  |  |  | Biologically favorable | 3 | 6 | 2 | 7 | 4 | 5 | 1 |
|  | Mean | 0.00 /5.91 | 0.00 /4.71 | 0.00 /4.69 | 0.00 /2.14 | 0.00 /4.23 | | 0.00 /4.08 | 0.11 /5.40 | Minimal change | 6 | 5 | 4 | 1 | 3 | 2 | 7 |
|  |  |  |  |  |  |  |  |  |  | Biologically favorable | 7 | 6 | 5 | 2 | 4 | 3 | 1 |
|  | D2% | –0.55 /9.76 | 0.00 /8.81 | 0.00 /9.78 | 2.08 /6.95 | 0.30 /7.36 | | 0.16 /7.94 | 0.00 /8.94 | Minimal change | 6 | 1 | 3 | 7 | 5 | 4 | 2 |
|  |  |  |  |  |  |  |  |  |  | Biologically favorable |  |  |  |  |  |  |  |
| OAR | D98% | 0.00 /4.26 | 0.34 /2.36 | 0.00 /4.46 | 0.45 /1.36 | 0.35 /2.10 | | –0.34 /6.42 | 1.31 /3.49 | Minimal change | 1 | 3 | 2 | 6 | 5 | 4 | 7 |
|  |  |  |  |  |  |  |  |  |  | Biologically favorable | 2 | 4 | 3 | 6 | 5 | 1 | 7 |
|  | Mean | 0.00 /43.81 | 2.99 /12.44 | 0.00 /41.69 | 0.37 /17.49 | 3.17 /15.36 | | 2.26 /16.27 | 0.00 /30.00 | Minimal change | 3 | 6 | 2 | 4 | 7 | 5 | 1 |
|  |  |  |  |  |  |  |  |  |  | Biologically favorable | 3 | 6 | 2 | 4 | 7 | 5 | 1 |
|  | D2% | 0.00 /72.53 | 5.64 /37.50 | 0.00 /75.05 | 0.16 /109.84 | 5.59 /47.04 | | 6.16 /57.51 | –0.39 /83.07 | Minimal change | 1 | 6 | 2 | 3 | 5 | 7 | 4 |
|  |  |  |  |  |  |  |  |  |  | Biologically favorable | 2 | 6 | 3 | 4 | 5 | 7 | 1 |
|  | | | | | | | Total score | | | Minimal change | 31 | 38 | 27 | 32 | 42 | 40 | 42 |
|  |  |  |  |  |  |  |  |  |  | Biologically favorable | 26 | 38 | 23 | 27 | 35 | 30 | 17 |
|  |  |  |  |  |  |  | Rank | | | Minimal change | 2 | 4 | 1 | 3 | 6 | 5 | 6 |
|  |  |  |  |  |  |  |  |  |  | Biologically favorable | 3 | 7 | 2 | 4 | 6 | 5 | 1 |

Supplementary Table 6. Spot weight distribution by beam configuration (Section 2).

C-shaped target; PTV3mm versus robust 3%/3 mm. Field-level values are averaged across the fields of each configuration.

| **Configuration** | **Fields** | **Energy layers PTV / Robust** | **CV PTV / Robust** | **ΔCV** | **Distal third (%) PTV / Robust** | **ΔDistal** |
| --- | --- | --- | --- | --- | --- | --- |
| 2Lat | 2 | 51.5 / 53.5 | 1.029 / 1.136 | +0.107 | 38.6 / 39.3 | +0.8 |
| 2AR | 2 | 41.5 / 43.0 | 1.114 / 1.147 | +0.033 | 57.4 / 52.7 | −4.7 |
| 2AO | 2 | 61.0 / 63.0 | 1.142 / 1.088 | −0.054 | 52.1 / 47.8 | −4.3 |
| 2PO | 2 | 60.5 / 62.0 | 0.893 / 0.973 | +0.080 | 30.7 / 30.4 | −0.3 |
| 3ALR | 3 | 44.7 / 46.3 | 1.097 / 0.954 | −0.143 | 51.8 / 46.5 | −5.3 |
| 4FB | 4 | 41.3 / 43.3 | 1.000 / 0.848 | −0.153 | 41.5 / 45.2 | +3.7 |
| 4FO | 4 | 60.8 / 62.5 | 1.158 / 0.985 | −0.173 | 42.6 / 44.1 | +1.5 |

Robust optimization used more energy layers in every configuration (Wilcoxon signed-rank, p = 0.016). ΔCV was inversely correlated with the number of fields (Pearson r = −0.864, p = 0.012). No consistent change in distal weight fraction was observed (p = 0.469). Abbreviations: CV, coefficient of variation.

Supplementary Table 7. Nominal-to-worst-case deviation in target LETd and RBE-weighted dose metrics by optimization strategy (Section 1).

Values are the most negative percentage change from the nominal plan across the full scenario set, averaged across the three uncertainty settings (1, 3, and 5). D98% and Dmean refer to the RBE-weighted dose. Worst-case values arose from the 5%/5 mm scenario in all cases.

| **Target** | **Optimization strategy** | **LETd98% low** | **LETd mean low** | **D98% low** | **Dmean low** |
| --- | --- | --- | --- | --- | --- |
| Rod-shaped | PTV-based | -14.3 | -13.3 | -32.8 | -6.0 |
|  | Robust translation | -29.1 | -15.5 | -51.2 | -6.7 |
|  | Robust density | -8.4 | -10.4 | -33.7 | -2.8 |
|  | Robust Trans&Dens | -8.8 | -10.1 | -23.5 | -2.1 |
| C-shaped | PTV-based | -11.7 | -15.0 | -77.6 | -6.2 |
|  | Robust translation | -20.8 | -18.2 | -84.1 | -7.8 |
|  | Robust density | -17.0 | -15.5 | -81.1 | -9.6 |
|  | Robust Trans&Dens | -16.7 | -13.6 | -65.8 | -6.3 |

Supplementary Table 8. Nominal-to-worst-case deviation in target LETd and RBE-weighted dose metrics by beam configuration (Section 2).

C-shaped target; PTV3mm versus robust 3%/3 mm. Values are the most negative percentage change from the nominal plan of the same configuration, across the full scenario set. D98% and Dmean refer to the RBE-weighted dose.

| **Plan** | **Optimization** | **LETd98% low** | **LETd mean low** | **D98% low** | **Dmean low** |
| --- | --- | --- | --- | --- | --- |
| 2Lat | PTV | -12.3 | -15.2 | -79.0 | -6.1 |
|  | Robust | -13.2 | -13.7 | -71.6 | -4.9 |
| 2AR | PTV | -20.1 | -15.8 | -68.6 | -10.2 |
|  | Robust | -20.2 | -13.1 | -44.9 | -4.8 |
| 2AO | PTV | -27.2 | -20.0 | -74.6 | -5.3 |
|  | Robust | -27.7 | -15.8 | -53.8 | -5.3 |
| 2PO | PTV | -18.3 | -9.3 | -61.8 | -8.8 |
|  | Robust | -27.1 | -6.9 | -51.9 | -8.4 |
| 3ALR | PTV | -13.8 | -15.7 | -58.5 | -9.8 |
|  | Robust | -14.1 | -13.2 | -37.2 | -5.1 |
| 4FB | PTV | -7.3 | -10.7 | -48.3 | -11.8 |
|  | Robust | -7.1 | -12.9 | -38.0 | -6.3 |
| 4FO | PTV | -0.5 | -10.5 | -60.2 | -8.4 |
|  | Robust | -0.6 | -13.9 | -48.3 | -4.8 |
